# Multiparametric Evaluation of Geriatric Patients Admitted to Intermediate Care: Impact on Geriatric Rehabilitation

**DOI:** 10.3390/diagnostics13182906

**Published:** 2023-09-11

**Authors:** Giuseppe Murdaca, Sara Banchero, Marco Casciaro, Francesca Paladin, Michele Tafuro, Fiammetta Monacelli, Alessio Nencioni, Roberta Bruschetta, Giovanni Pioggia, Gennaro Tartarisco, Sebastiano Gangemi

**Affiliations:** 1 Department of Internal Medicine, University of Genoa, IRCCS Ospedale Policlinico San Martino, 16132 Genoa, Italy; sarabanchero@hotmail.it; 2 IRCCS Ospedale Policlinico San Martino, 16132 Genoa, Italy; puell-a@hotmail.it (F.P.); micheletafuro@gmail.com (M.T.); fiammetta.monacelli@unige.it (F.M.); alessio.nencioni@unige.it (A.N.); 3 Department of Medical Sciences, University Hospital of Messina, 98125 Messina, Italy; marco.casciaro@unime.it; 4 Section of Geriatrics, Department of Internal Medicine and Medical Specialties (DIMI), University of Genoa, 16132 Genoa, Italy; 5 Institute for Biomedical Research and Innovation (IRIB), National Research Council of Italy (CNR), 98164 Messina, Italy; roberta.bruschetta@irib.cnr.it (R.B.); giovanni.pioggia@cnr.it (G.P.); gennaro.tartarisco@cnr.it (G.T.); 6 Department of Engineering, Università Campus Bio-Medico di Roma, Via Alvaro del Portillo 21, 00128 Rome, Italy; 7 School and Operative Unit of Allergy and Clinical Immunology, Department of Clinical and Experimental Medicine, University of Messina, Via Consolare Valeria 1, 98125 Messina, Italy; sebastiano.gangemi@unime.it

**Keywords:** elderly, geriatric, rehabilitation, intermediate care, multiparametric, statistics, cognitive impairment, dementia

## Abstract

Optimizing the functional status of patients of any age is a major global public health goal. Rehabilitation is a process in which a person with disabilities is accompanied to achieve the best possible physical, functional, social, intellectual, and relational outcomes. The Intermediate Care Unit within the O.U. of Geriatrics and Gerontology of the San Martino Hospital in Genoa is focused on the treatment and motor reactivation of patients with geriatric pathologies. The objective of this study was to identify which factor, among the characteristics related to the patient and those identified by the geriatric evaluation, had the greatest impact on rehabilitation outcomes. Our findings revealed significant correlations between the Barthel Index delta, the 4AT Screening Test, and the number of drugs taken. This association highlights the potential benefits of medication management in enhancing the overall well-being and functional abilities of frail older adults, despite the literature suggesting that polypharmacotherapy is associated with a reduction in functional status and an increase in mortality. These findings underscore the significance of a multidimensional geriatric assessment. Refining and optimising these multidisciplinary approaches is the objective of a more effective geriatric rehabilitation strategy.

## 1. Introduction

Functional status refers to an individual’s capability to perform routine daily activities essential for fulfilling basic needs, habitual roles and maintaining health and well-being. Biological factors, cognitive impairment, mood disorders, and other variables can influence an individual’s functional status [1]. Optimizing the functional status of individuals, regardless of age, represents a pivotal global public health objective [2]. In particular, the ageing population contributes to an escalating number of individuals facing a decline in functional status due to increasing multimorbidity (the coexistence of multiple chronic diseases) and geriatric syndromes (typical conditions of advanced age with non-specific manifestations reflecting dysfunction across various organism functions [3]. This situation elevates the risk of disability, indicating a diminished capacity to engage with the social environment and adversely affecting the ability to conduct daily activities, potentially leading to dependence [4].

Physical exercise has emerged as a fundamental strategy for preserving physiological reserves, yielding favourable effects on diverse aspects including musculoskeletal strength, neural health, respiratory and cardiovascular systems, body composition, and metabolism [5].

Rehabilitation, as defined by the Italian Ministry of Health in 2019, is a process in which individuals with disabilities are supported in attaining optimal autonomy across physical, functional, social, intellectual, and relational dimensions while acknowledging their limitations.

Similarly, geriatric rehabilitation, as defined by the Dutch National Aged Care Program in 2017, constitutes a multidisciplinary integrated care approach aimed at restoring the functional status of frail elderly individuals after acute events or in cases of functional challenges.

In the geriatric setting, rehabilitation often focuses on fall prevention, postoperative rehabilitation, and management of chronic conditions such as arthritis, dementia, and stroke. Geriatric rehabilitation also improves the quality of life of elderly patients through patient and family education, emotional support, and promotion of a healthy lifestyle.

Geriatric rehabilitation can be defined as a non-specific therapeutic approach aimed at the sick elderly person at risk of disability or specific when it is implemented on an already disabled patient. The former approach seeks to intervene on the functional loss that follows the interaction between ageing and disease, and the latter aims to recover and maintain the maximum possible level of functional autonomy in the disabled elderly patient population.

Multidisciplinarity is indispensable in geriatric rehabilitation to address the intricate needs of frail patients [6]. Key players in geriatric rehabilitation include specialised geriatric physicians, responsible for executing multidimensional assessments to identify the diverse factors contributing to rehabilitation outcomes. Multidimensional geriatric assessment serves as a multidisciplinary diagnostic process designed to objectively define an elderly patient’s health status, tailor treatment plans to individual needs, and forecast prognoses.

This study was conducted at the Intermediate Care Unit within the Geriatrics and Gerontology Department of San Martino Hospital in Genoa, which specialises in functional recovery, clinical stabilisation and therapeutic optimisation for post-acute phase patients or those with chronic conditions yet to attain hospital discharge criteria. The unit concentrates on treating and reactivating motor functions in individuals grappling with geriatric pathologies, notably encompassing post-stroke patients, those with Parkinsonism, chronic obstructive pulmonary disease, heart disease, hip fractures, and cognitive impairments.

## 2. Materials and Methods

A total of 50 patients admitted to the Intermediate Care Unit of the O.U. of Geriatrics and Gerontology of the San Martino Hospital in Genoa were recruited during hospitalisation between November 2022 and March 2023. Of these, 22 were male and 28 were female, aged between 70 and 95 years.

The collected data included demographic information, several clinical scales at admission as input, and the clinical assessment of the Barthel index at two time points: admission (T0) and discharge (T1) as outcome. The demographic and clinical characteristics of all participants are summarised in Table 1 and presented as mean ± SD and median (min-max).

The main objective of this study was to identify which factor, among the characteristics related to the patient and among the characteristics identified by the geriatric evaluation, had the greatest impact on rehabilitation outcomes.

The success or failure of geriatric rehabilitation was measured by determining the Barthel Index at entry and discharge within the multidimensional assessment, as explained later.

The factors considered in our study included:

− Gender: according to the current literature, gender could play a role in rehabilitation outcomes [7]. Indeed, the 2019 Global Burden of Disease Study (GBD) suggests that while men and women have a similar prevalence of conditions benefiting from rehabilitation, women appear to have higher YLD (years lived with disability) than men. However, there are limited data in the literature regarding the rehabilitation differences between men and women, especially in the geriatric field.
− Age: the patient’s chronological age at the time of hospitalisation is considered in our study with the aim of determining whether it could have an impact on the rehabilitation outcome. At the moment, according to our knowledge, there are few studies that have examined this association. The focus of the current literature seems to be the association between cognitive impairment typical of old age and its impact on learning motor skills and, consequently, its influence on geriatric rehabilitation [8].
− Days of hospitalisation: the days of hospitalisation were considered for the evaluation of geriatric rehabilitation because an increase in the length of hospitalisation is typically associated with a higher prevalence of complications, especially in elderly patients [9]. However, a reduction in the number of days of hospitalisation is correlated with a shorter duration of rehabilitation treatment.
− Pre-hospitalization Activities of Daily Living (ADL): ADL is a score that examines activities of daily living that are essential for living in a social world as they enable survival and basic well-being, such as bathing, going to the toilet, dressing, and eating [10]. Prehospitalization ADL detection must be carried out with the patient and relatives to gain as much as possible a truthful picture of autonomy at home. It allows the objectification of the pre-admission functional status as an essential clinical tool for identifying rehabilitation objectives, studying a personalised path to recover pre-admission autonomy as much as possible, and monitoring the success of the physiotherapy treatment.
− Barthel Index at the time of admission and discharge: this is a score that establishes the patient’s degree of independence. It consists of 10 items that examine common daily activities. Each item is assigned a score whose sum indicates the patient’s degree of autonomy in carrying out daily life activities [11]. In our study, it was used to determine the success or failure of the rehabilitation therapy performed during hospitalisation.
− Clinical Frailty Scale (CFS) at the time of admission: CFS is a frailty tool that assesses specific domains, including comorbidity, functional status, and cognition, to generate a frailty assessment score ranging from 1 (very fit) to 9 (terminally ill). A score of >4 points indicates frailty [12]. Frailty is a multifactorial syndrome determined by the reduction of physiological functional reserve and the ability to resist stressful conditions, which causes vulnerability to adverse events such as hospitalisation and disability [13]. It is characterized by reduced homeostatic reserves and clinically significant vulnerability, concomitant age-related loss of autonomy, and a high disease burden [14]. Identification of frail older adults at high risk of adverse outcomes is crucial for subsequent resource planning and targeted interventions. In our study, CFS was used to objectively assess the patient’s degree of frailty, as multiple reviews confirmed that CFS is a valid tool for the study of frailty [15]. The short- and long-term outcomes of geriatric rehabilitation can be influenced by the frailty status of patients [16].
− Hand Grip Strength Test upon admission: This test measures the maximum isometric force exerted by the muscles of the upper limb. It is performed using a dynamometer that records each person’s strength. It is a simple and reliable measure of maximum voluntary muscle strength. It is an important tool for diagnosing sarcopenia and is widely used as a single indicator to represent overall muscle strength; it can predict not only muscle mass and physical activity but also the incidence of chronic disease, nutritional status, quality of life, independence of daily living, length of hospital stay, and even mortality [17].
− Anticholinergic cognitive burden score (ACB score) at the time of admission: anticholinergic drugs work by blocking the neurotransmitter acetylcholine in the central or peripheral nervous system and have different actions depending on the site [18]. The neurotransmitter acetylcholine is implicated in several processes that are impaired during delirium, such as attention, sleep, and memory, allowing the hypothesis that the anticholinergic load could be involved in the pathogenesis of delirium [19]. It is important to study the anticholinergic burden in patients in geriatric rehabilitation because delirium appears to be associated with less successful rehabilitation outcomes [20]. All medications taken by patients upon entry into the ward were classified according to the 2012 update of the ACB score.
− Number of medications at admission: polypharmacotherapy, defined as the regular use of at least five medications, is common in the elderly and increases the risk of adverse medical outcomes [21]. The identification of the number of drugs used in chronic therapy by the patient in geriatric rehabilitation allows us to study the effect of polypharmacy on the rehabilitation outcome. In fact, a strong bidirectional relationship between polypharmacy and physical function has been demonstrated [22]. According to recent literature, polypharmacy leads to an increased risk of frailty [23] and risk of falling [21], thus linking polypharmacy to an uncertain outcome of physiotherapy treatment.
− Mini Nutritional Assessment Short-Form (MNA-SF) at admission: malnutrition in the elderly results from numerous changes in physiological function with age, for example, decreased food intake, less physical exercise, reduction of intestinal absorption, mental problems, etc., [24]. Since the causes of malnutrition are numerous, it is necessary to use a screening tool that examines multiple factors, such as the Mini Nutritional Assessment or its simplified form, i.e., the Mini Nutritional Assessment Short-Form, which consists of a questionnaire made up of six items. Both the complete and reduced forms of MNA are effective in identifying the presence of malnutrition in elderly patients [25]. In the path of physiotherapy functional recovery, it is essential to recognise and treat malnutrition, as this appears to be linked to sarcopenia, depression, cognitive impairment, increased risk of falls, delayed immune response, increased risk of infection, and, more generally, increases the incidence of frailty [24].
− 4AT Screening Test (4AT) upon admission: This is a tool for assessing the presence of delirium or brief cognitive impairment that is widely used internationally in clinical practise and research [26]. Delirium is an acute onset confusional state characterised by an altered level of attention and self-awareness in the environment (arousal), with circadian fluctuations [27]. It is a geriatric syndrome of vulnerable and frail patients. The 4AT consists of four items: the first evaluates the level of vigilance, there is also an orientation test, an attention test (months backwards test), and an item that determines the acute change or the fluctuating course of the state of conscience [28].
− Cumulative Illness Rating Scale (CIRS) at the time of admission: this scale was developed for the assessment of physical pathology and examined 14 independent body domains [29]. Our study is based on the evaluation of the severity index, which results from the average of the scores of the first 13 categories and whose maximum obtainable score is 5, with the aim of comparing the rehabilitation results with an evaluation of the severity of the pathologies of the individual.

### Statistical Analyses

Our first inclination was to apply machine learning analysis to the collected data [30]. After the first approach, we noticed that traditional statistics performed better. The normal distribution of data was assessed for all variables using the Shapiro–Wilk test. In cases where the normality assumption was not met, we measured the skewness of the distribution to apply the appropriate transformation method according to the type of normality violation. Specifically, we applied the square root transformation for moderately skewed variables and the inverse transformation for severely skewed ones. Moreover, the sign of skewness was considered for the correction.

The difference between the Barthel Index at discharge and at admission (T1 and T0) was used as an output variable to investigate which admission variables predicted the change in patients’ level of autonomy. In particular, Pearson’s correlation coefficient was initially used to measure the relationship between the Barthel Index Delta and other clinical variables at T0, and then highly correlated variables were employed in a multiple linear regression model to predict the outcome.

In addition, the nonparametric Mann–Whitney–Wilcoxon (MWW) test was used to verify that there was no significant difference in Barthel Index Delta between genders.

Subsequently, the patients were categorised into two groups based on their Barthel Index Delta: Low (below or equal to 10) and High (above 10). Univariate analysis was performed to examine potential significant differences between the two groups. Specifically, we used the parametric *t*-test for normally distributed continuous variables, the Mann–Whitney–Wilcoxon (MWW) test for not normally distributed continuous variables, and the chi-square test for categorical variables.

Statistically significant variables in the univariate analysis were then introduced in a multivariate logistic regression model. All statistical analyses were performed using R version 4.3. The *p* value was considered significant at <0.05. Leave-one-out cross-validation was used to evaluate logistic regression model performance. Principal component analysis was used to visualise how the two groups were discriminated by the selected variables.

## 3. Results

The results of the Shapiro–Wilk test are reported in Table 2. Values < 0.05 indicate that the data are not normally distributed. Variables that violate the assumption of normality are indicated with an asterisk. The skewness values of non-normally distributed variables and the corresponding mathematical transformations employed to correct them are reported in Table 3. By retesting the Shapiro–Wilk test on the transformed variables, we obtained the results shown in Table 4, revealing an improvement in the distribution for Hospital Stay and CIRS variables. However, in the case of Handgrip, as there was a deterioration of the distribution, subsequent analyses were conducted considering the original variable. Pearson’s correlation test between Barthel Index Delta and each variable at admission evidenced a significant positive correlation with Drugs Number (r = 403, *p* = 0.004) and a significant negative correlation with 4AT Screening Test (r = −342, *p* = 0.015) with the outcome. All test results are reported in Table 5. The MWW test of Barthel Index Delta between genders showed no significant difference (U = 302, *p* = 0.913).

The fitted multilinear regression model was:

Barthel Index Delta = 0.1884 − 4.2060 × 4AT Screening Test + 1.8373 × Drugs Number

The overall regression was statistically significant (R^2^ = 0.23, F_(2,47)_ = 6.91, *p* = 0.002).

Drugs Number significantly predicted Barthel Index Delta (β = 1.83, *p* = 0.01).

No high significance was found for 4AT Screening Test (β = −4.21, *p* = 0.05).

Details of the model output are reported in Table 6.

**Figure 1:**
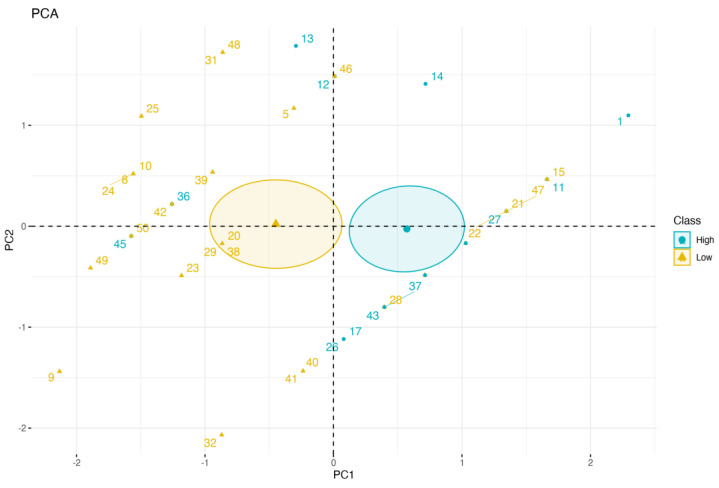
The visualization of two Barthel classes (High vs. Low) using the first two components of PCA.

Results of univariate analyses, reported in Table 7, showed a significant difference between the two groups for variables Drugs Number and 4AT Screening Test, confirming what was previously found on continuous outcome. The logistic regression model trained using these two features achieved an accuracy of 70% in classifying the two groups (Low vs. High Barthel Index variation). The overall performances of the model are reported in Table 8. Additionally, Figure 1 displays the outcomes of principal component analysis (PCA), which serves as a powerful visualisation tool to gain insights into the data’s structure and relationships. The PCA plot, which explains the overall variability in the dataset, visually represents these relationships, showing the distribution of individuals along the first two principal components. It helps to identify two clusters of Barthel classes, enabling a deeper understanding of the underlying characteristics.

## 4. Discussion

The analysed data and performed tests explored the relationship between the Barthel Index delta, representing the change in functional independence over time, and several variables. Pearson’s correlation, which measures the strength and direction of the linear relationship between two continuous variables, revealed significant positive correlations between the Barthel Index Delta and the number of drugs taken. This moderate positive relationship indicates that as the number of medications taken by individuals increases, there is a tendency for a higher Barthel Index delta, signifying improved functional independence over the course of hospitalisation. While this observation suggests that medications might positively influence functional independence, it contradicts the existing literature associating polypharmacotherapy with adverse effects, drug interactions, increased anticholinergic burden, and mortality risk. Therefore, this correlation should not be interpreted causally because other factors could influence the association.

In addition, a significant negative correlation was observed between the Barthel Index Delta and the 4AT Screening Test. This moderate negative relationship suggests that as the presence of delirium increases, as assessed by the 4AT Screening Test, there is a tendency for a lower Barthel Index Delta, indicating declining functional independence during hospitalisation. Delirium can impede an individual’s ability to perform daily activities, leading to increased reliance on support and assistance. Notably, no substantial differences in rehabilitation performance were found between male and female patients, despite the recognised role of gender differences in ageing. The absence of gender-related disparities in our data contrasts with expectations derived from recent gender medicine concepts, which emphasise metabolic distinctions between sexes and variations in life expectancy.

Results from the multilinear regression model confirm significant positive associations between the number of drugs and outcome. Conversely, the negative relationship between the 4AT Screening Test and outcome remain substantial but lacks statistical significance. It is important to note that correlation analysis and multivariate regression models, while both shed light on variable associations, entail slightly nuanced interpretations. Correlation analysis provides a broad measure of linear associations between variables without delving into causality. Multivariate regression is context-specific, assuming cause-and-effect relationships between independent variables and the outcome. These relationships are influenced by the context and inherent nature of the variables. Moreover, the inclusion of multiple variables in a model can introduce intricate interactions that impact the significance of individual variables. In addition, smaller datasets may struggle to detect significant relationships in a multivariate model, even when significant correlations exist. Nevertheless, despite the linear model not definitively confirming the significance of the 4AT screening test, the results consistently highlight its substantial negative impact. This conclusion is further substantiated by the Mann–Whitney test conducted on the Barthel Index, firmly establishing the test’s significant influence on the outcome.

The Barthel Index is frequently used to measure functional independence in older adults, whereas frailty plays a significant role in rehabilitation outcomes because of reduced physiological reserves and heightened stress vulnerability. Frail individuals often score lower on the Barthel Index because of challenges in daily activities. Accordingly, a negative correlation between the Barthel Index and frailty is anticipated. The 4AT, a cognitive assessment tool used to screen for delirium and cognitive impairment in the elderly, suggests that frail older adults with cognitive impairment and delirium risk require tailored rehabilitation strategies that address cognitive and functional needs. Successful delirium prevention and treatment encompass the identification of causative factors, drug prescribing, environmental adjustments, and drug therapies such as antipsychotics.

Rehabilitation substantially contributes to an enhancement in functional outcomes and promotion of independence in frail older adults. The Barthel Index frequently serves as an outcome measure that reflects rehabilitation intervention effectiveness. Tailored rehabilitation programmes encompassing physical therapy, occupational therapy, and cognitive training cater to the specific needs of frail individuals optimising functional capabilities and overall well-being.

The correlations observed illustrate the intricate interplay between functional independence, cognitive status, medication use, and frailty in rehabilitation contexts. These findings underscore the necessity for a comprehensive, multidimensional approach to frail older adults’ care, which integrates cognitive status, medication management, and functional rehabilitation requirements. Furthermore, these correlations should be considered within the broader context of individual patient characteristics, encompassing comorbidities, social support, and rehabilitation goals when devising personalised care plans. Healthcare professionals must incorporate these factors to enhance frail older adults’ functional outcomes and quality of life.

## 5. Conclusions

In conclusion, this study underscores the indispensable role of multidisciplinary approaches in geriatric rehabilitation to address the complexity of improving functional outcomes and well-being in frail patients. Geriatric specialists play a pivotal role in conducting comprehensive geriatric assessments, identifying factors influencing rehabilitation intervention outcomes.

Focused on functional recovery, clinical stabilisation and therapeutic optimisation for patients in the post-acute phase or with conditions requiring further stabilisation, our study emphasises the importance of addressing geriatric pathologies. We aimed to determine which components of the examined geriatric syndrome had the greatest impact on rehabilitation outcomes.

Our findings revealed significant correlations between the Barthel Index delta, the 4AT Screening Test, and the number of drugs taken. This association highlights the potential benefits of medication management in enhancing overall well-being and functional abilities in frail older adults. However, the counterintuitive nature of these correlations compared with the existing literature suggests limitations, such as our study’s small sample size (composed of 50 patients), necessitating further investigation in a larger geriatric population.

Conversely, the presence of delirium, as assessed by the 4AT Screening Test, is associated with declining functional independence, likely due to reduced active participation in physiotherapy, heightened fall risk, and cognitive impairment. Our study’s objective is to heighten the awareness of clinicians treating elderly patients during rehabilitation to early delirium identification, as it influences the success of physiotherapy treatment. Preventive measures, including prescribing techniques and environmental and pharmacological interventions, can mitigate delirium effects.

Our unit places particular emphasis on the treatment and motor reactivation of individuals with geriatric pathologies, including post-stroke patients, those with Parkinsonism, chronic obstructive pulmonary disease, heart disease, hip fracture, and cognitive impairment; however, the negative correlation with the presence of delirium indicates the need to broaden geriatric rehabilitation. This should be undertaken with the purpose of motor reactivation in conjunction with a multidisciplinary rehabilitation approach aimed at delaying both functional and cognitive decline and reducing psychological and behavioural disorders by acting on neuroplasticity and functional reserve [31]. Integrated rehabilitation techniques can include occupational therapy, with the aim of maintaining autonomy in daily life, stimulating residual functional abilities, and developing compensation strategies [32]. Cognitive-oriented treatments have also been developed with the common goal of improving or maintaining cognitive functions and include cognitive training, cognitive rehabilitation, and cognitive stimulation [33].

In summary, the observed correlations emphasise the significance of comprehensive geriatric assessment and collaborative approaches. Refinement and optimisation of these multidisciplinary strategies constitute the aim of an effective geriatric rehabilitation strategy.

## Figures and Tables

**Table 1:** Demographic and clinical characteristics of enrolled participants. Data are expressed as mean **±** SD and median (range). The Barthel index is the dependent variable.

Measure	Descriptive Statistics
	Mean ± SD
	Median (Range)
Gender (M/F)	(22/28)
Age (Years)	81.3 ± 5.9
	81.5 (70–95)
Hospital Stay	16.18 ± 7.07
	15.00 (3–38)
Clinical Frailty Scale (CFS)	6.74 ± 0.72
	7 (4–8)
Hand Grip Strength Test	15.19 ± 6.3
	13 (1–31)
Anticholinergic cognitive burden score (ACB score)	2 ± 2.2
	1 (0–8)
Drugs Number	8.64 ± 2.26
	9 (3–14)
Mini Nutritional Assessment Short Form (MNA-SF)	8.24 ± 2.48
	9 (2–13)
4AT Screening Test (4AT)	0.96 ± 1.28
	0 (0–4)
Cumulative Illness Rating Scale (CIRS)	2.24 ± 0.41
	2.17 (1.38–3.50)
Activities of Daily Living (ADL)	5.12 ± 1.35
	6 (0–6)
Admission Barthel (T0)	26.8 ± 19.4
	20 (5–95)
Discharge Barthel (T1)	40.2 ± 23.3
	32.5 (5–100)
Delta Barthel	13.4 ± 12.1
	10 (−5–55)

**Table 2:** Descriptive statistics and the Shapiro–Wilk test of variables before transformations.

							Shapiro–Wilk	
	N	Mean	Median	SD	Min	Max	W	*p*
Sex	50	1.560	2.00	0.501	1	2	0.632	<0.001 *
Age	50	81.300	81.50	5.905	70	95	0.965	0.143
Hospital Stay	50	16.180	15.00	7.073	3	38	0.924	0.003 *
CFS	50	6.740	7.00	0.723	4	8	0.703	<0.001 *
Handgrip	50	15.190	13.00	6.304	1.00	31.00	0.930	0.005 *
ACB Score	50	2.000	1.00	2.204	0	8	0.819	<0.001 *
Drugs Number	50	8.640	9.00	2.257	3	14	0.972	0.278
MNA	50	8.240	9.00	2.479	2	13	0.963	0.123
4AT Screening Test	50	0.960	0.00	1.277	0	4	0.750	<0.001 *
CIRS	50	2.244	2.17	0.409	1.38	3.50	0.949	0.031 *
ADL	50	5.120	6	1.350	0	6	0.703	<0.001 *

* significative value.

**Table 3:** Skewness value and corresponding heuristic transformation applied to correct the normality violation for each variable.

Variable	Skewness	Transformation
Hospital Stay	0.35	sqrt(x)
CFS	−1.82	1/(max(x + 1) − x)
Handgrip	−0.23	sqrt(max(x + 1) − x)
ACB Score	0.16	sqrt(x)
4AT Screening Test	0.50	sqrt(x)
CIRS	0.41	sqrt(x)
ADL	−3.3	1/(max(x + 1) − x)

**Table 4:** Descriptive statistics and the Shapiro–Wilk test of variables after transformations.

							Shapiro–Wilk	
	N	Mean	Median	SD	Min	Max	W	*p*
Sex	50	1.560	2.00	0.501	1	2	0.632	<0.001
Age	50	81.300	81.50	5.905	70	95	0.965	0.143
Hospital Stay	50	16.180	15.00	7.073	3	38	0.924	0.321
CFS	50	6.740	7.00	0.723	4	8	0.703	<0.001
Handgrip	50	15.190	13.00	6.304	1.00	31.00	0.930	<0.001
ACB Score	50	2.000	1.00	2.204	0	8	0.819	< 0.001
Drugs Number	50	8.640	9.00	2.257	3	14	0.972	0.278
MNA	50	8.240	9.00	2.479	2	13	0.963	0.123
4AT Screening Test	50	0.960	0.00	1.277	0	4	0.750	<0.001
CIRS	50	2.244	2.17	0.409	1.38	3.50	0.949	0.125
ADL	50	5.120	6	1.350	0	6	0.703	<0.001

**Table 5:** Results of the Pearson correlation test between each independent variable at admission and the Barthel Index Delta.

	Barthel Index Delta	
	Pearson’s r	*p*-Value
Age	−0.053	0.715
Hospital Stay	0.124	0.389
CFS	−0.095	0.511
Handgrip	0.263	0.065
ACB Score	−0.028	0.845
Drugs Number	0.403	0.004 *
MNA	0.027	0.855
4AT Screening Test	−0.342	0.015 *
CIRS	−0.124	0.391
ADL	0.237	0.098

* significative value.

**Table 6:** Results of the multilinear regression model.

	Estimate	Std. Error	t Value	Pr (>|t|)
Intercept	0.1884	6.7579	0.028	0.9779
4AT Screening Test	−4.2060	2.1206	−1.983	0.0532
Drugs Number	1.8373	0.7098	2.588	0.0128

Residual standard error: 10.9 on 47 degrees of freedom. Multiple R-squared: 0.2272, adjusted R-squared: 0.1943. F-statistic: 6.91 on 2 and 47 df, *p*-value: 0.002341.

**Table 7:** Results of univariate analysis between low and high Barthel index delta. The *t*-test was used for normally distributed continuous variables, the Mann–Whitney–Wilcoxon (MWW) test for not normally distributed continuous variables, and the Chi-square test for categorical variables.

		Statistics	gdl	*p*
Sex	$\chi^{2}$	0.574	1	0.449
Age	*t*-Student	0.0191	48.0	0.985
Hospital Stay	*t*-Student	0.0755	48.0	0.940
CFS	Mann–Whitney–Wilcoxon	279	48.0	0.482
Handgrip	Mann–Whitney–Wilcoxon	249	48.0	0.248
ACB Score	Mann–Whitney–Wilcoxon	273	48.0	0.487
Drugs Number	*t*-Student	2.5152	48.0	0.015 *
MNA	*t*-Student	−0.6029	48.0	0.549
4AT Screening Test	Mann–Whitney–Wilcoxon	188	48.0	0.009 *
CIRS	*t*-Student	−0.8127	48.0	0.420
ADL	Mann–Whitney–Wilcoxon	248	48.0	0.188

* significative value.

**Table 8:** Results of the logistic regression model.

Accuracy	F1-Score	Precision	Recall	ROC AUC
0.70	0.70	0.78	0.64	0.70

## Data Availability

The data were provided in the text.
